# Levosimendan displays anti-inflammatory effects and decreases MPO bioavailability in patients with severe heart failure

**DOI:** 10.1038/srep09704

**Published:** 2015-04-13

**Authors:** Matti Adam, Sven Meyer, Henning Knors, Anna Klinke, Ulf K. Radunski, Tanja K. Rudolph, Volker Rudolph, Joshua M. Spin, Philip S. Tsao, Angelika Costard-Jäckle, Stephan Baldus

**Affiliations:** 1 Stanford University, Division of Cardiovascular Medicine, Stanford, CA, USA; 2 University of Groningen, University Medical Center Groningen, Department of Cardiology, Groningen, The Netherlands; 3 European Medical School Oldenburg-Groningen, Heart Center Oldenburg, Department of Cardiology, Oldenburg, Germany; 4 University of Hamburg, Heart Center, Department of Cardiovascular Medicine, Hamburg, Germany; 5 University of Cologne, Heart Center, Department of Cardiology and Cologne Cardiovascular Research Center, Cologne, Germany

## Abstract

Treatment of decompensated heart failure often includes administration of levosimendan. Myeloperoxidase (MPO) is released during polymorphonuclear neutrophil (PMN) degranulation, and mediates dysregulation of vascular tone in heart failure. We evaluated the effects of levosimendan-treatment on MPO in patients with acute decompensation of chronic heart failure over a one week course. Plasma MPO levels were significantly decreased after levosimendan treatment (from 252.1 ± 31.1 pmol/l at baseline to 215.02 ± 27.96 pmol/l at 6 h, p < 0.05). *Ex vivo* incubation of whole blood with levosimendan decreased MPO release after PMN-stimulation (8.2 ± 1.4-fold increase at baseline vs. 6.0 ± 1.1-fold increase with levosimendan). MPO levels also significantly correlated with diastolic blood pressure over the time course. In a multivariate linear model, the main contributor to systolic, diastolic and mean blood pressure was level of PMN elastase. MPO contributed only in heparin-treated patients, suggesting a more significant role for endothelial-bound MPO than for circulating MPO or elastase with respect to blood pressure regulation. We here provide the first evidence that levosimendan treatment inhibits MPO release by PMNs in decompensated heart failure patients. This mechanism may regulate endothelial function and vascular tone in heart failure patients.

Heart failure (HF) is a significant cause of morbidity in Western society, affecting approximately 1–2% of the adult population. The prevalence is considered to be 8.4% for patients older than 75 years, whereas the risk of developing new onset heart failure is 0.2/1000 person-years in those aged 45–55 years, and increases up to 12.4/1000 person-years for those over age 85. Five year mortality is high, ranging from 45% in women up to 59% in men, and accounting for 55,000 annual deaths in the US alone^1^,^2^,^3^.

The majority of patients hospitalized for acute heart failure have worsening chronic heart failure, rather than *de novo* symptoms. Published data provide evidence for polymorphonuclear neutrophils (PMN) activation and myeloperoxidase (MPO)-release in patients with severe HF^4^,^5^,^6^, with higher MPO levels found in patients with more aggressive disease. MPO is a redox-activated hemoprotein, and is abundant in PMNs. While MPO is mainly stored inside azurophilic granules of PMNs, it is also expressed in monocytes and tissue-associated macrophages. Upon secretion, it accumulates along the endothelium and in the subendothelial space. Beyond MPO's bactericidal properties, its role as a mediator and contributor in vascular inflammatory processes involved in coronary artery disease and HF has been recently appreciated^7^,^8^,^9^. Endothelial nitric oxide (NO) bioavailability is reduced by active MPO^8^, and outcomes in HF patients are adversely affected by decreased endothelial NO bioavailability^10^,^11^. Moreover, vascular tone is profoundly altered by MPO^12^, and dysregulation of vascular tone and afterload in patients are major pathobiological features in HF^13^,^14^,^15^.

For patients showing clinical signs of acute peripheral hypoperfusion due to HF, the current European Society of Cardiology guidelines suggest levosimendan as a viable treatment option to help reverse the negative inotropic effects of pre-existing oral beta-blocker treatment. Levosimendan, is a calcium sensitizer and opens K^+^-channels, but also has complex pleiotropic effects^16^. It has been shown that levosimendan not only increases cardiac output^17^ but also may improve endothelial function^18^ and suppress lipid peroxidation, protein oxidation and nitrosative stress^19^.

The present study assessed the effects of levosimendan on PMN function and activation by analysis of MPO and elastase levels as indicators of neutrophil degranulation in patients with acute heart failure. Further, we analyzed the correlation of PMN activation with blood pressure regulation and vascular tone in these patients.

## Methods

### Study outline

Twenty-five consecutive acute heart failure patients admitted to the Department of General and Interventional Cardiology at the University Heart Center Hamburg were enrolled. Patients referred to the unit with signs of acute heart failure were evaluated for cardiac function and signs of acute peripheral hypoperfusion as well as for co-morbidities and other potential causes for acute decompensation. All patients underwent transthoracic echocardiography to verify low cardiac output, defined as left ventricular ejection fraction below 30% (Simpson method). Patient inclusion criteria consisted of: age >17 years, heart failure NYHA class III and IV, or ACC/AHA class D, acute worsening of heart failure with signs of peripheral hypoperfusion, clinical indication for treatment with levosimendan (not determined by an study investigator), left ventricular ejection fraction below 30% determined by echocardiography or cardiac MRI). Exclusion criteria comprised: severe impairment of renal function (creatinine clearance <30 ml/min), severe hepatic dysfunction (bilirubin >2.0 mg/dl), systemic inflammatory disorders, overt infection, malignancy, autoimmune disorders, and concomitant treatment with intravenous vasoactive agents, such as inotropes or catecholamines.

Mean arterial pressure was calculated as diastolic blood pressure plus 1/3 of pulse pressure (the difference between systolic and diastolic blood pressure). Right heart catheterization was performed if indicated per standard-of-care (to verify diagnosis or plan further treatment) within 24 hours before levosimendan treatment (twelve out of twenty-five patients).

The study was performed in accordance with the Declaration of Helsinki and approved by the Board of Physicians Ethics Committee Hamburg. All patients provided informed consent.

### Levosimendan treatment

Levosimendan solution (0.025 mg/ml) was prepared using a 5 ml vial containing 12.5 mg of levosimendan concentrate (Orion Oyj Pharma, Finland) diluted in 500 ml sterile 5% glucose (B. Braun, Germany). Administration was performed via a central venous line at a constant rate of 21 ml/hour over 24 hours with no loading dose. If during administration a clinically relevant decrease in blood pressure occurred, as judged by the treating physician, the infusion was temporarily halted for a maximum of 1 hour, and then re-started at a 50% decreased rate.

### Blood sample collection

Blood was collected at baseline (immediately before levosimendan administration) and at 1, 6, 24, and 48 hours, as well as 1 week after treatment initiation. Blood samples were processed immediately, and 2 ml of separated plasma were pelleted and stored at −80°C pending further processing.

### Laboratory measurements

MPO was quantitated using the CardioMPO kit (Cleveland Heart Lab) according to manufacturer's instructions. Elastase immunoassay was performed using a PMN-Elastase ELISA kit (IBL International). Plasma nitrite was analyzed using a Nitrate/Nitrite Fluorometric Assay Kit (Cayman chemicals) without performing nitrate reduction.

Additionally, the following biomarkers were measured in plasma: C-reactive protein (CRP; nephelometric immunoassay, Siemens for high sensitive CRP), N-terminal prohormone of brain natriuretic peptide (NT-proBNP; LOCI-Test, Siemens) and interleukin-6 (antibody-ECLIA, Roche). The following data were recorded from clinical routine laboratory tests performed at the clinical central laboratory, University Hospital Hamburg: differential blood count, CRP, AST, ALT, GGT, bilirubin and creatinine.

### In vitro analysis of whole blood

EDTA-blood was collected from healthy human subjects and incubated with levosimendan for 6 hours (500 ng/ml) at room temperature. Samples were subsequently stimulated with 20 μl of HBSS +0.25% BSA (control), or 20 μl of 100 nM PMA (phorbol 12-myristate 13-acetate), or 1 μM fMLP (N-formyl-Met-Leu-Phe). Stimulation was performed for 45 minutes at room temperature. Plasma was collected through centrifugation (1000 x g, 10 minutes) and stored at −80°C until further analysis.

### Statistical analysis

All calculations were made with SPSS 17.0 (Statistical Package for Social Science, Chicago) and GraphPad Prism 6.01. Normal distribution tested by Kolmogorov-Smirnov-Test led to pairwise testing with Students unpaired t-test. Otherwise the Mann-Whitney rank sum test was performed. Linear mixed-model regression was used for multiple comparisons, and post-hoc Fisher's Least Significant Difference (LSD) tests were performed. Differences between timepoints of levosimendan administration (for MPO, for example) were calculated using a linear mixed model with inclusion of intersubject effects and timepoint as repeated measurements, the specific plasma marker as dependent variable, and post-hoc LSD testing.

Correlations were calculated using a Pearson product-moment correlation coefficient if variables were interval-scaled and normally distributed. Otherwise, rank correlation using a Spearman's coefficient was performed.

Multivariable testing was performed using a general linear model including systolic and diastolic blood pressure as dependent variables, and all markers of inflammation that were significantly correlated with blood pressure as factors or co-variates (out of MPO, elastase, nitrite, leukocyte count, granulocyte count, CRP, interleukin-6). Inter-subject effects were calculated via partial eta squared.

A p value of <0.05 was considered statistically significant. Data are presented as mean ± SEM or median ± interquartile range if data were not normally distributed or not interval scaled.

## Results

### Patient Characteristics

Table 1 shows the baseline patient characteristics. The mean age was 58.4 ± 2.6 years, and 28% of patients were suffering from ischemic heart disease. The mean pulmonary wedge pressure was 20.3 ± 3.9 mmHg, and 56% of patients were in NYHA Class IV functional status.

### Markers of leukocyte function/PMN-activation and degranulation

**Figure 1:**
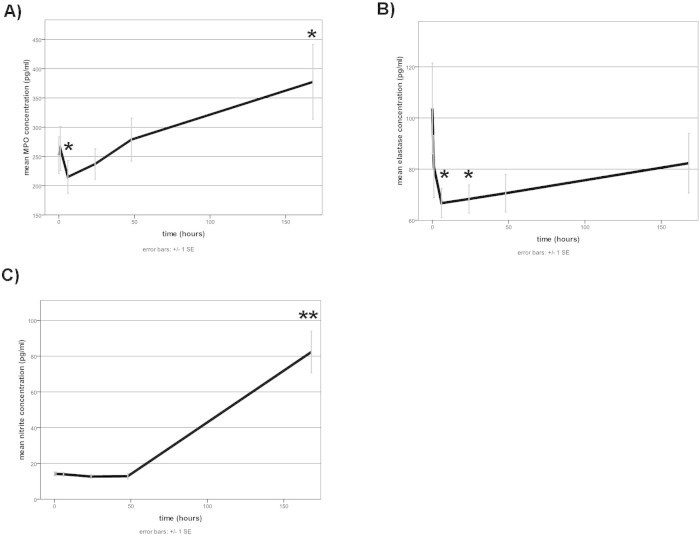
Effects of levosimendan on neutrophilic markers and markers of NO-production in plasma. Assessment of plasma markers at each specific time point after levosimendan application (hours) for (A) MPO-concentration, (B) elastase-concentration and (C) nitrite-concentration. Data displayed are for all available 25 patients (0–24 hours), 19 patients (48 hours) and 18 patients (168 hours). * = p ≤ 0.05, ** = p ≤ 0.01 versus timepoint 0 hours. Level of significance was determined using a mixed linear model with posthoc LSD test.

Levosimendan treatment led to significantly decreased MPO levels at 6 hours (from baseline 252.1 ± 31.1 to 215.02 ± 27.96 pmol/l at 6 h, p < 0.05; figure 1 A) and significantly increased levels over the next week (to 377.4 ± 15.4 pmol/l, p < 0.05), while elastase levels were decreased at 6 and 24 h (from baseline 103.2 ± 17.7 ng/ml to 81 ± 12.1 ng/ml at 6 h and 66.7 ± 5.7 ng/ml at 24 h, p < 0.05; figure 1 B), but showed no significant change after 2 or 7 days. Mean plasma nitrite concentration as a marker of NO-production^20^ was not different for the first 2 days but did show a highly significant increase after 7 days (from baseline 14.2 ± 1.1 μM to 82.3 ± 11.7 μM at 7 days, p < 0.01; figure 1 C).

### Association analysis of blood pressure and inflammatory markers

As we were specifically interested in the association between PMN activation and vascular tone, we evaluated the correlations between systolic, diastolic, and mean blood pressure, and nitrite levels and markers of leukocyte activity (elastase, MPO, granulocyte count, leukocyte count) as well as inflammation (CRP, interleukin-6) in our patients (table 2A). Plasma MPO, elastase and leukocyte count all showed significant correlations with systolic, diastolic or mean blood pressure. Multivariate linear regression analysis with systolic, diastolic and mean blood pressure as dependent variables revealed that plasma elastase and leukocyte count contributed significantly to the final model, with the highest partial eta for elastase (table 2B).

It has been previously reported that administration of heparin significantly increases levels of plasma MPO in patients. Mechanistically, this is due to competitive mobilisation of MPO from extracellular matrix proteins or endothelial cells by heparan sulfate-based glycosaminoglycans^8^. However, we identified no significant effect of heparin administration on plasma MPO or elastase levels in our patient cohort at any time point. To evaluate whether the amount of vessel-bound MPO contributes to blood pressure regulation in heart failure patients, we performed the above correlation and multivariate analyses by stratifying patients in groups *with* (n = 9) or *without* (n = 16) heparin therapy during levosimendan-administration (table 3 and 4). In patients without heparin administration, only leukocyte count contributed to the final model of blood pressure, while the model of blood pressure regulation in heparin-treated patients included interleukin-6 and MPO, but not elastase.

### Ex vivo evaluation of PMN-activation and degranulation in whole blood

**Figure 2:**
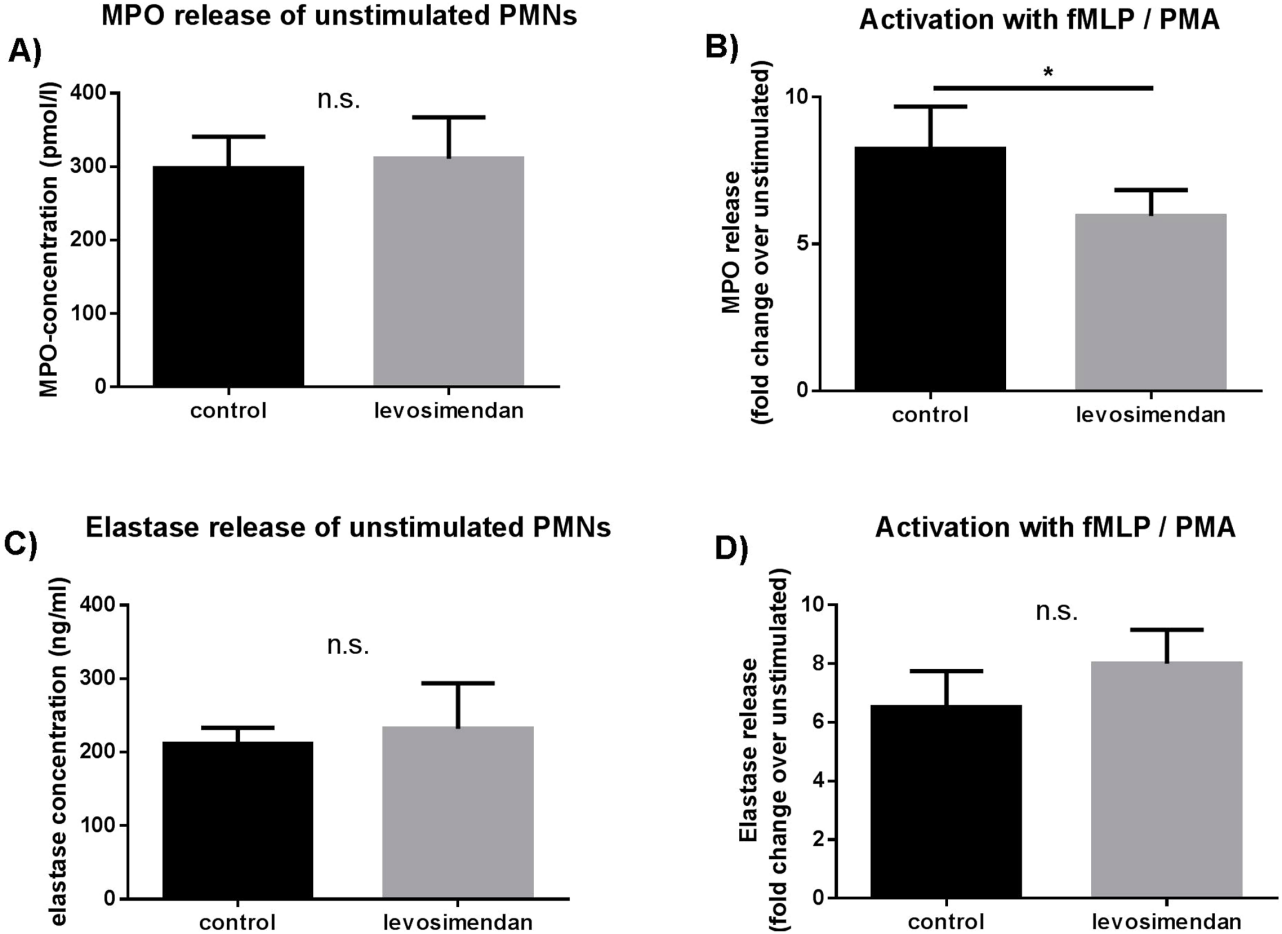
*Ex vivo* effects of Levosimendan on MPO release in whole blood. Whole blood was taken from healthy controls and subsequently incubated with 500 ng/ml Levosimendan for 6 hours at room temperature. If indicated, samples were stimulated with 20 μl of 100 nM PMA (phorbol 12-myristate 13-acetate) and 1 μM fMLP (N-formyl-Met-Leu-Phe) for 45 minutes on room temperature. (A) MPO-concentration after Levosimendan incubation alone, n = 22. (B) Fold change of MPO-concentration of stimulated (fMLP/PMA) over non-stimulated cells with and without Levosimendan incubation, n = 10. * = p ≤ 0.05, ** = p ≤ 0.01 versus other group. Level of significance was determined using a Student's T - test.

To investigate the direct effects of levosimendan on PMN-activation, we incubated whole blood *ex vivo* with levosimendan at a concentration of 500 ng/ml. Levosimendan alone did not have a direct effect on MPO release from leukocytes in whole blood, but after PMN-activation via fMLP/PMA, levosimendan significantly decreased MPO-release vs. no levosimendan (8.2 ± 1.4-fold change vs. 6 ± 0.9-fold change; Figure 2 A-B).

### Markers of inflammation and heart failure

**Figure 3:**
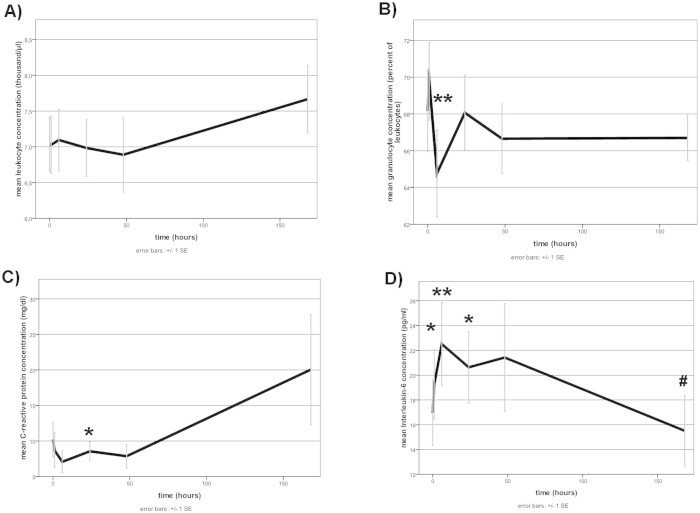
Effects of Levosimendan on markers of inflammation in plasma. Assessment of plasma markers at each specific time point after levosimendan application (hours) for (A) leukocyte concentration, (B) relative granulocyte count, (C) CRP-concentration, (D) Interleukin-6 concentration. Data displayed are for all available 25 patients (0–24 hours), 19 patients (48 hours) and 18 patients (168 hours). * = p ≤ 0.05, ** = p ≤ 0.01 versus timepoint 0 hours. Level of significance was determined using a mixed linear model with posthoc LSD test.

**Figure 4:**
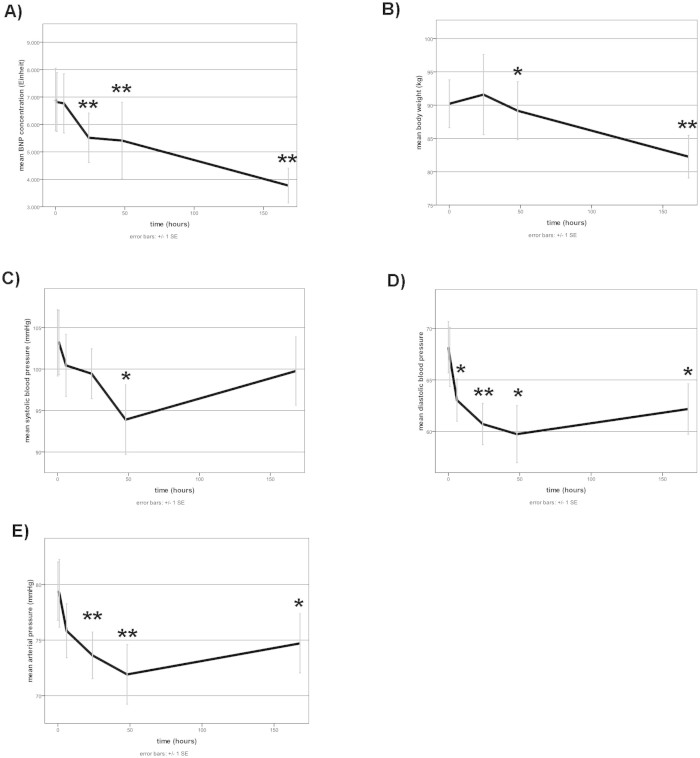
Effects of Levosimendan on heart failure markers and blood pressure in plasma. Assessment of plasma markers at each specific time point after levosimendan application (hours) for (A) NT-proBNP (B) body weight, (C) systolic blood pressure, (D) diastolic blood pressure, (E)mean arterial pressure. Data displayed are for all available 25 patients (0–24 hours), 19 patients (48 hours) and 18 patients (168 hours). * = p ≤ 0.05, ** = p ≤ 0.01 versus timepoint 0 hours. Level of significance was determined using a mixed linear model with posthoc LSD test.

While leukocyte count did not vary over the week-long time course (Fig. 3A), there was a significant drop in relative PMN levels at 6 hours after levosimendan application (64.78 ± 2.37% vs. 68.17 ± 2.22% at baseline, Fig. 3B. Serum levels of CRP showed a significant decrease at 24 h (10.2 ± 2.4 vs. 8.55 ± 1.4 mg/dl, Fig. 3C). Interleukin-6 plasma levels were significantly elevated in the first 24 h after levosimendan treatment and decreased over the time course (Fig. 3D). NT-proBNP decreased from 6907.9 ± 1142.0 pg/ml to 3772.5 ± 634.4 pg/ml, and patient body weight dropped from 90.2 ± 3.6 kg to 82.3 ± 3.2 kg over the 7 days (Fig. 4A and B).

While severe hypotension is considered a known complication necessitating stoppage of levosimendan-treatment, this did not occur in any of our patients, although in 2 patients inotropic therapy was escalated and further data were excluded. Nevertheless, we could show statistically significant decreases in systolic, diastolic and mean blood pressure in patients treated with levosimendan (Fig. 4 C and D).

## Discussion

In this prospective, observational study of 25 patients with severe heart failure, levosimendan-treatment reduced markers of cardiac decompensation, like body weight or NTpro-BNP as previously described^21^. Intriguingly, neutrophil elastase and MPO plasma concentrations (markers of neutrophil activation) were decreased 24 h after treatment with levosimendan. *Ex vivo* incubation of EDTA-blood with levosimendan decreased MPO release after PMN-stimulation. Additionally, there was a significant correlation between neutrophil elastase and systolic, diastolic as well as mean blood pressure over the complete time course, MPO correlated significantly with diastolic blood pressure. In a multivariate linear model, elastase remained a contributor to a model including systolic, diastolic and mean blood pressure as dependent variables. A similar contribution could be shown for MPO, but only in heparin-treated patients, suggesting a more significant role for endothelial-bound MPO than for circulating MPO or circulating elastase with respect to blood pressure correlation. Plasma nitrite levels as a surrogate for overall NO-bioavailability were significantly increased 1 week after treatment with levosimendan.

While levosimendan has been shown to have anti-oxidative, anti-inflammatory and anti-apoptotic effects, its immunmodulatory effects are not completely understood and may be an important mechanism to improve abnormal neurohormonal responses in heart failure^22^,^23^,^24^. A recent study by Hasslacher et al.^25^ has shown that levosimendan inhibits the release of reactive oxygen species from stimulated PMNs *in vitro* and from PMNs derived from heart failure patients. Our study demonstrates that levosimendan incubation of whole blood decreases MPO release after stimulation *ex vivo*, underlining the immunmodulatory effects of levosimendan. Additionally, our *ex vivo* data imply a systemic effect of levosimendan on PMN activation with lower levels of MPO 24 hours after treatment. This is in agreement with published data showing improved endothelial function 24 hours after levosimendan treatment^18^, as leukocyte activation and especially MPO activity can significantly impair endothelial function^8^. Avgeropoulou and colleagues have found a decrease in MDA, a marker of oxidative stress, at day 5 after levosimendan treatment^26^, which supports our hypothesis of diminished PMN degranulation and MPO activity after levosimendan treatment, as MPO activity and MDA-levels usually correlate well^27^.

Further, in a placebo-controlled study in patients with severe heart failure, Parissis and colleagues^19^ reported a significant increase in markers of oxidative and nitrosative stress like MDA, nitrotyrosine and protein carbonyls in the placebo group 48 hours after treatment which did not occur in the levosimendan-treated group. This indicates an anti-oxidative and anti-nitrosative effect of levosimendan, as nitrotyrosine formation is downstream of MPO activity, with MPO-generated nitrogen dioxide (NO_2_•) strongly contributing to nitrotyrosine formation^7^. These anti-inflammatory effects might in part be explained by inhibition of MPO-release by levosimendan.

Most intriguingly, we identified a significant increase in MPO levels at 7 days after levosimendan. This augmentation in MPO-concentration occurred without a coincident increase in elastase levels, indicating an underlying mechanism separate from PMN degranulation. Several other explanations may apply. Former studies have shown that a release of MPO from the endothelial glycocalyx is possible, allowing the vessel wall to achieve higher NO-availability and improved flow-mediated dilation^8^. The significant increase in plasma nitrite, a sustainable marker of NO production by eNOS, which occurred simultaneously with the increase in MPO, might suggest that MPO was released from the endothelium, increasing NO-bioavailability. More detailed studies are needed to explore this, as we can only speculate that increases in MPO and nitrite plasma levels are causally connected.

Increased nitrite plasma levels can also be a marker of macrophage activation^28^, but we did not find elevated markers of inflammation corresponding with nitrite levels. In fact, there were no significant increases in interleukin-6, granulocyte count, leukocyte count or CRP at 1 week after levosimendan treatment.

In porcine endothelial cells, levosimendan causes an increase in NO-concentration by activation of eNOS via MAP-kinases like p38, AKT and ERK^29^. However, we would expect that effects of levosimendan on endothelial cells and eNOS would be more pronounced at earlier timepoints, leaving the increase of nitrite levels in our patient group unexplained.

Adamopoulos et al. showed anti-inflammatory effects of levosimendan on day 3 after levosimendan in heart failure patients with regard to TNF-alpha, interleukin-6, soluble Fas and soluble Fas-ligand^24^. While we cannot provide information on TNF-alpha or Fas, we did measure IL-6. In our patient population, which had distinctly higher IL-6 and NT-proBNP levels than in Adamopoulus et al., we found an increase in IL-6 concentration at 6, 24 and 48 hours after the beginning of levosimendan treatment, but then a subsequent decline vs. baseline with a significant decrease from 12 h to 7 d after treatment. This decline over days has been previously observed in 29 patients with severe heart failure at day 5 after levosimendan treatment^26^. Ischemia and subsequent reperfusion have been shown to increase IL-6 release from rat cardiomyocytes^30^. Therefore our IL-6 peak might be at least in part explained by early levosimendan-induced hemodynamic improvements. Furthermore, in our population, the initial increase in IL-6 is not reflected by CRP curve progression, even though both markers positively correlate over all patients and the complete time course (r = 0.384, p < 0.01). Intriguingly, there was a clear opposition in IL-6 and CRP-curve shape during the study period. This was unexpected, as IL-6 is considered a main inducer for acute-phase-protein production, which includes CRP. It has been shown before that both markers are elevated during acute cardiac decompensation^31^, and subsequently decrease with improvement of that condition, but may have different kinetics^32^. Therefore, a therapeutic approach to HF with levosimendan might resemble a condition in which IL-6 is increased but CRP will not necessarily follow.

We additionally evaluated parameters of PMN activation and inflammation that were associated with blood pressure in a multivariate regression model. While our analysis does not provide causal insights, we utilized systolic, diastolic and mean blood pressure as an indirect estimate of endothelium-dependent vasoconstriction, as blood pressure correlates with endothelial function^33^,^34^,^35^ and mean arterial pressure is an indicator for general vascular resistance and perfusion pressure^36^,^37^. Therefore, it is of importance that elastase and MPO contributed to final models of blood pressure. Intriguingly, the contribution of MPO exceeded the contribution of elastase to the final model in the heparin-treated patients, as elastase was not significantly associated with blood pressure in these patients. This is coincident with the observation of an increased correlation coefficient between MPO and diastolic blood pressure in patients with heparin administration compared to no heparin or all patients (heparin: R = 0.349, p < 0.05; no heparin: 0.042, n.s.; all patients: 0.179, p < 0.05). The administration of heparin may have mobilized a portion of endothelial-immobilised MPO, which could then be quantified, and which otherwise would have remained unmeasured and ‘hidden’ on the endothelium.

Since we found that endothelial-bound MPO and PMN activation are significantly correlated with blood pressure regulation in HF patients treated with levosimendan, this implies a direct influence of blood pressure regulation on leukocyte and especially PMN activation and endothelial MPO deposition. On the other hand, given the inhibitory effect of levosimendan on MPO-release in whole blood *ex vivo*, and after 24 hours *in vivo*, inhibition of PMN function through levosimendan might contribute to better endothelial-dependent vascular function with more NO bioavailability and lower blood pressure. A direct effect on PMNs would imply less MPO deposition on the endothelial layer^38^ and former work from our lab has already shown that MPO-activity in PMNs is inversely correlated with flow-mediated dilation, with MPO administration impairing myocardial perfusion.

We acknowledge that our work has certain limitations. The implementation of a randomized control group (randomization of levosimendan application vs. no levosimendan application) raised ethical concerns. Therefore we do not have a sufficient untreated control group, and can only compare changes over the time course of levosimendan application, but not to patients that have improved without levosimendan treatment. Further, the correlation and regression analyses we performed do not necessitate causal connection, and in general show moderate strength.

Nevertheless, our data suggest that levosimendan has inhibitory effects on MPO release which might be beneficial for vascular endothelial function, contributing to blood pressure regulation in heart failure patients. Given the general state of neurohormonal activation which accompanies acute heart failure decompensation, the direct effects of levosimendan on MPO release and PMN activation have likely been under-recognised.

## Author Contributions

M.A. designed the study, collected patient data, performed experiments, wrote and edited the manuscript. S.M. designed the study, collected patient data and edited the manuscript. H.K. collected patient data and performed experiments. A.K. designed the study, performed experiments and edited the manuscript. U.K.R. collected patient data. J.M.S. reviewed and edited the manuscript. T.K.R., V.R., P.S.T. and A.C.J. reviewed and edited the manuscript and provided supervision. S.B. designed the study, reviewed and edited the manuscript and provided supervision.

## Figures and Tables

**Table 1:** Patient characteristics

Characteristic	Number	Percentage
Gender (male vs. Female)	22/3	88%
Etiology (ischemic vs. Non ischemic)	7/18	28%
Number of patients at timepoint 0 h	25	100%
Number of patients at timepoint 1 h	25	100%
Number of patients at timepoint 6 h	25	100%
Number of patients at timepoint 24 h	25	100%
Number of patients at timepoint 48 h	19	76%
Number of patients at timepoint 168 h	18	72%
Mortality	0	0%
age (years)	58.4 ± 2.6	
BMI (kg/m^2^)	27.7 ± 4.7	
Presence of edema	13	52%
Dyspnea NYHA III	11	44%
Dyspnea NYHA IV	14	56%
Ejection fraction (Echocardiography) <30%	25	100%
ACE-inhibitors/ARBs	18	72%
Beta-blockers	19	76%
Diuretics	20	80%
Digoxins	2	8%
Heparins	13	52%
Cumarins	11	44%
Statins	9	36%
Heart rate at rest (bpm)	76 ± 6	
Systolic blood pressure at rest (mmHg)	103 ± 8	
Diastolic blood pressure at rest (mmHg)	68 ± 5	
Aspartate aminotransferase (U/l)	48.1 ± 10.7	
Alanine aminotransferase (U/l)	61.6 ± 16.4	
Gamma – glutamyl transferase (U/l)	136.1 ± 24.2	
Total bilirubine (mg/dl)	1.0 ± 0.2	
Creatinine mg/dl (mg/dl)	1.5 ± 0.1	
C-reactive protein (mg/dl)	5.9 ± 6.1	
RAP - mean (mmHg)	9.4 ± 3.5	
RVP - systolic (mmHg)	45.9 ± 9.6	
RVP - diastolic(mmHg)	8.3 ± 2.9	
PAP - systolic (mmHg)	45.8 ± 9.6	
PAP - diastolic (mmHg)	21.5 ± 4	
PAP - mean (mmHg)	30.8 ± 5.7	
PCWP - mean (mmHg)	20.3 ± 3.9	
Cardiac index (l/min/m^2^)	1.9 ± 0.6	

BMI = body mass index, RAP = right atrial pressure, RVP = right ventricular pressure, PAP = pulmonary artery pressure, PCWP = pulmonary capillary wedge pressure.

**Table 2:** Association analysis of blood pressure and inflammatory markers, all patients

A) Correlation analysis – coefficients of correlation shown						
	MPO	Elastase	Leukocyte count	Granulocyte count	Interleukin-6	Systolic/diastolic BP
BP systolic	0.089 (n.s.)	0.293**	0.245**	−0.111 (n.s.)	0.155 (n.s.)	0.611**
BP diastolic	0.179*	0.402**	0.076 (n.s.)	−0.153 (n.s.)	−0.026 (n.s.)	0.611**
BP mean	0.167 (p = 0.06)	0.393**	0.172*	−0.156 (n.s)	0.019 (n.s)	0.866**/0.931**
	CRP					
BP systolic	−0.068 (n.s.)					
BP diastolic	−0.034 (n.s.)					
BP mean	−0.053 (n.s)					

B) Multivariate analysis		
Parameter	Significance	Partial eta-square
**Elastase**	**<0.001**	**0.141**
**Leukocyte count**	**<0.01**	**0.080**
MPO	n.s	

Correlation analysis (A) of systolic, diastolic and mean blood pressure with markers of neutrophil activation and inflammation (MPO, elastase, leukocyte count, granulocytic count, Interleukin-6, CRP). Coefficients of correlation are shown. Correlations were calculated using a Pearson product-moment correlation coefficient if variables were interval-scaled and normally distributed (MPO, elastase, leukocyte count, granulocytic count, Interleukin-6). Otherwise, rank correlation using Spearman's coefficient was performed (CRP).

Significant correlations were included into a multivariate linear model including systolic, diastolic and mean blood pressure as dependent variables (B).

* = p ≤ 0.05, ** = p ≤ 0.01. BP systolic = systolic blood pressure, BP diastolic = diastolic blood pressure, BP mean = mean arterial blood pressure, MPO = Myeloperoxidase, CRP = C-reactive protein.

**Table 3:** Association analysis of blood pressure and inflammatory markers, patients without heparin medication

A) Correlation analysis – coefficients of correlation shown						
	MPO	Elastase	Leukocyte count	Granulocyte count	Interleukin-6	Systolic/diastolic BP
BP systolic	0.089 (n.s.)	0.293**	0.285**	−0.232*	−0.282**	0.747**
BP diastolic	0.042 (n.s.)	0.219*	0.076 (n.s.)	−0.247*	−0.116 (n.s.)	0.747**
BP mean	0.076 (n.s.)	0.177 (n.s.)	0.123 (n.s.)	−0.257*	−0.206 (n.s.)	0.907**/0.957**
	CRP					
BP systolic	−0.422**					
BP diastolic	−0.202 (n.s.)					
BP mean	−0.304**					

B) Multivariate analysis		
Parameter	Significance	Partial eta-square
**Leukocyte count**	**<0.01**	**0.173**
Elastase	p = 0.056	0.083
Granulocyte count	n.s.	
Interleukin-6	n.s.	
CRP	n.s.	

Correlation analysis (A) of systolic, diastolic and mean blood pressure with markers of neutrophil activation and inflammation (MPO, elastase, leukocyte count, granulocytic count, Interleukin-6, CRP). Coefficients of correlation are shown. Significant correlations were included into a multivariate linear model including systolic, diastolic and mean blood pressure as dependent variables (B). for details see also Table 2.

* = p ≤ 0.05, ** = p ≤ 0.01. BP systolic = systolic blood pressure, BP diastolic = diastolic blood pressure, BP mean = mean arterial blood pressure, MPO = Myeloperoxidase, CRP = C-reactive protein.

**Table 4:** Association analysis of blood pressure and inflammatory markers, patients with heparin medication

A) Correlation analysis– coefficients of correlation shown						
	MPO	Elastase	Leukocyte count	Granulocyte count	Interleukin-6	Systolic/diastolic BP
BP systolic	0.138 (n.s.)	0.366**	0.075 (n.s.)	−0.013 (n.s.)	0.482**	0.469**
BP diastolic	0.349*	0.522**	0.055 (n.s.)	−0.058 (n.s.)	0.066 (n.s.)	0.469**
BP mean	0.279 (p = 0.055)	0.530**	0.092 (n.s.)	−0.54 (n.s.)	0.271 (n.s.)	0.815**/0.904**
	CRP					
BP systolic	0.480**					
BP diastolic	0.230 (n.s.)					
BP mean	0.432**					

B) Multivariate analysis		
Parameter	Significance	Partial eta-square
**Interleukin-6**	**<0.01**	**0.282**
**MPO**	**<0.05**	**0.160**
Elastase	n.s.	
CRP	n.s.	

Correlation analysis (A) of systolic, diastolic and mean blood pressure with markers of neutrophil activation and inflammation (MPO, elastase, leukocyte count, granulocytic count, Interleukin-6, CRP). Coefficients of correlations are shown. Significant correlations were included into a multivariate linear model including systolic, diastolic and mean blood pressure as dependent variables (B). for details see also Table 2.

* = p ≤ 0.05, ** = p ≤ 0.01. RR syst = systolic blood pressure, RR diast = diastolic blood pressure, MPO = Myeloperoxidase, CRP = C-reactive protein.
